# An unexpected new species of the genus *Pseudopoda* (Araneae, Sparassidae, Heteropodinae) from the Western Ghats in India

**DOI:** 10.3897/zookeys.577.7848

**Published:** 2016-04-05

**Authors:** Peter Jäger, Siddharth Kulkarni

**Affiliations:** 1 Arachnology, Senckenberg Research Institute, Senckenberganlage 25, 60325 Frankfurt am Main, Germany; 2 Hemi Terrace Bldg, Near Ellora Palace, Balajinagar, Pune- 411043, Maharashtra, India

**Keywords:** Taxonomy, Huntsman Spiders, morphology

## Abstract

A new species of the genus *Pseudopoda* is described from India: *Pseudopoda ashcharya* **sp. n.** Males are characterised by the absence of the conductor and females are unique within the genus in having the lateral lobes of their epigyne fused. The systematic relationship of the new species is discussed referring to its isolated occurrence in the Western Ghats.

## Introduction

Almost all members of the subfamily Heteropodinae as well as almost all species of the genus *Pseudopoda* exhibit a membranous conductor (Jäger 2001, 2002). Jäger et al. (2015) described the first *Pseudopoda* species without a conductor. *Pseudopoda wu* Jäger, Li & Krehenwinkel 2015 shows a small non-sclerotised patch at its tegulum, most likely the rest of an otherwise reduced conductor homologue. Jäger (2015) described five species from the Nat Ma Taung in Myanmar lacking a conductor entirely. Logunov and Jäger (2015) described another species without conductor from Vietnam: *Pseudopoda ohne*. Males of the present new species lack a conductor and females show unique characters as well. The species was found in the Western Ghats and far away from other *Pseudopoda* populations. This geographically unexpected finding is described as a new species and its systematic background is discussed.

## Material and methods

The examined spiders are preserved in 70 % denatured ethanol, samples for molecular analyses are kept in 99.5 % pure ethanol. Examination and drawings were carried out with a Leica MZ 16 stereomicroscope with camera lucida attachment. Female copulatory organs were dissected and the sclerotised internal duct system was cleared in 96% DL-lactic acid (C_3_H_6_O_3_). All measurements are in millimetres [mm]. Opisthosoma length means the length without petiolus and spinnerets. Leg formula, leg spination pattern and size classes follow Jäger (2001). Palp and leg lengths are listed as: total (femur, patella, tibia, metatarsus, tarsus). Arising points of tegular appendages in males are described as clock-positions of the left pedipalp in ventral view. In schematic illustration of the internal duct system the blind ending (glandular) appendage is marked with “T”, the copulatory orifice with a circle, and the end of the fertilisation duct in direction of the *uterus externus* with an arrow. As in Jäger (2005: 88), slit sensilla close to the epigyne are generally considered as descriptive character. Colouration is described from specimens in ethanol.

Elevation is given in metres [m]. Maps were produced with DIVA-GIS version 7.5.0.

Abbreviations used in the text: dRTA – dorsal part/branch of RTA, DS – dorsal shield of prosoma, mya – million years ago, OS – Opisthosoma, PJ – numbers represent subsequent numbers of Sparassidae examined by the first author, RTA – retrolateral tibial apophysis, SD – serial number of tissue samples for molecular analyses, vRTA – ventral part/branch of RTA, I–IV – referring to leg numbers.

Museum collections (with curators): BNHS – Bombay Natural History Society, Mumbai, India (Rahul Khot), SMF – Senckenberg Museum, Frankfurt, Germany (Julia Altmann, Peter Jäger).

## Results

### *Pseudopoda* Jäger, 2000

#### Pseudopoda ashcharya sp. n.

Taxon classificationAnimaliaAraneaeSparassidae

http://zoobank.org/173F36B2-BC0B-4656-901B-29D79680A268

Figs 1–9 , 10–17 , 18

##### Type material.

Holotype male, India, Maharashtra State, Devrukh, 17.068°N, 73.626°E [180 m elevation], leaf litter, 30 August 2013, PJ 3630, SD 1221 (SMF). Paratypes: 1 female, India, Maharashtra State, Guravwadi, 16.876°N, 73.645°E [100 m elevation], leaf litter, 2 September 2013, PJ 3631, SD 1222 (SMF). 1 male, India, Maharashtra State, Guravwadi, 16.876°N, 73.645°E [100 m elevation], leaf litter, S. Kulkarni leg., by hand, 15 August 2015, Sp. 316 (BNHS).

##### Etymology.

The species name is taken from the Hindi, “ashcharya” meaning “surprise”, referring to the unexpected occurrence of the genus *Pseudopoda* in the Western Ghats, more than 1400 km away from the closest congener; term in apposition.

##### Diagnosis.

**Figures 1–9.**
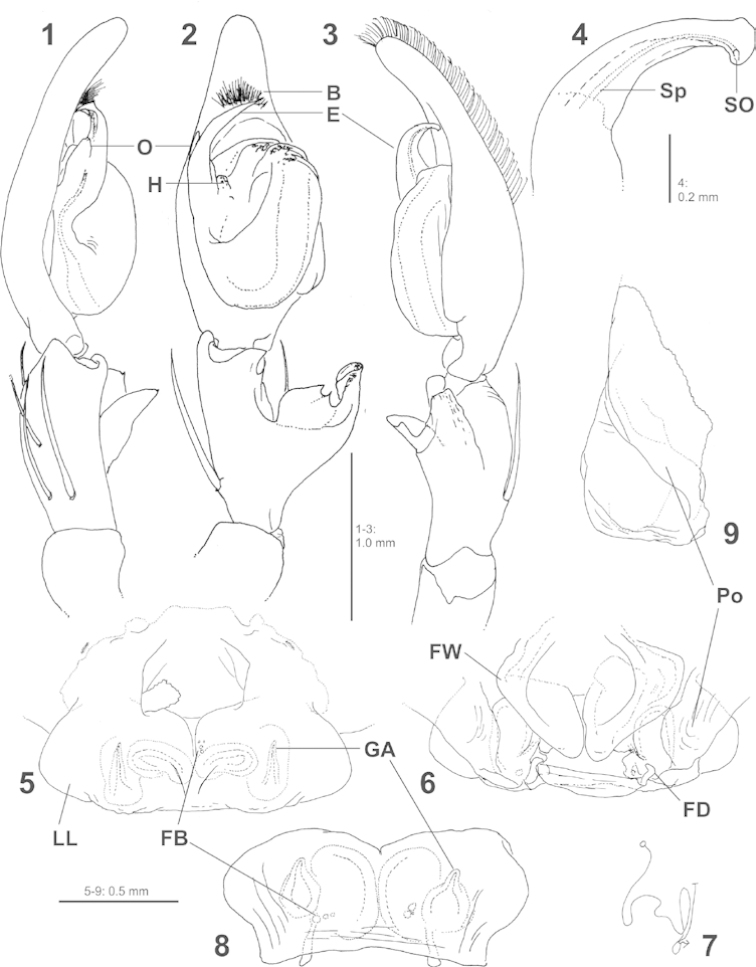
*Pseudopoda ashcharya* sp. n. from India, copulatory organs of male (**1–4** holotype) and female (**5–9** paratype). **1–3** Left male palp (**1** prolateral **2** ventral **3** retrolateral) **4** Embolus, retrolatero-proximal **5** Epigyne, ventral **6** Vulva, dorsal **7** Schematic course of internal duct system, dorsal **8** Epigyne, posterior **9** Epigyne, lateral. B brush of setae close to embolus tip
E embolus
FB fusion bubbles
FD fertilisation duct
FW first winding of internal duct system
GA glandular appendage
H hump at base of embolus
LL lateral lobe
O embolic outgrowth
Po sublobal pockets
Sp spermophor
SO spermophor opening.

Small Heteropodinae, body length male 8.4, female 8.3. Males similar to those of *Pseudopoda* species described in Jäger (2015), *Pseudopoda ohne* Logunov & Jäger 2015 and *Pseudopoda wu* in having the conductor entirely reduced, most similar to *Pseudopoda pingu* Jäger 2015, *Pseudopoda wamwo* Jäger 2015 and *Pseudopoda martinschuberti* Jäger 2015 in having the embolus roughly sickle-shaped in ventral view, but can be distinguished from these species by the mesal embolic outgrowth (Figs 1–4). Females may be recognised by the medially fused lateral lobes, the unique pointed glandular appendages and by the sublobal pockets (Figs 5–9).

##### Description.

Male (holotype). DS length 4.3, width 3.8, anterior width 1.9, OS length 4.1, width 2.1. Eyes: diameters AME 0.22, ALE 0.35, PME 0.27, PLE 0.33; interdistances AME–AME 0.14, AME–ALE 0.05, PME–PME 0.17, PME–PLE 0.37, AME–PME 0.37, ALE–PLE 0.33, clypeus height at AME 0.49, at ALE 0.40. Spination: palp: 131, 11(small, distal)1, 2101; legs: femur I–II 323, III 322, IV 331; patella I–IV 101; tibia I–IV 2026; metatarsus I–II 1014, III 2025, IV 3036. Metatarsus IV ventrally with double row of bristles along entire length and with patch of bristles instead distal spine, I–III with scopula, without bristles. Leg formula: 2(14)3. Measurements of palp and legs: palp 6.0 (2.0, 0.8, 1.1., -, 2.1), leg I 19.7 (5.2, 2.1, 5.8, 4.9, 1.7), leg II 20.4 (5.5., 2.1, 6.0, 5.1, 1.7), leg III 15.8 (4.4, 1.7, 4.5, 3.9, 1.3), leg IV 19.7 (5.5, 1.6, 5.2, 5.7, 1.7). Promargin of chelicerae with 3 teeth, retromargin with 4 teeth; cheliceral furrow with ca. 15 denticles in patch close to anterior teeth; chelicerae with 1 bristle close to retromargin of fang base.

Palp as in diagnosis (Figs 1–4). Cymbium slender, with dorsal scopula in distal half, retrolateral bulge with small, proximad hump. RTA arising proximally to mesally, vRTA with 2 small pointed apices, dRTA with blunt end. Spermophor running submarginally retrolaterally, narrowing prolaterally when entering the embolus. Embolus arising from 9- to 10-o’clock-position from tegulum, with small hump at its base centrally, its widened tip situated close to a dense brush of setae at the proximal part of cymbium’s tip.

**Figures 10–17.**
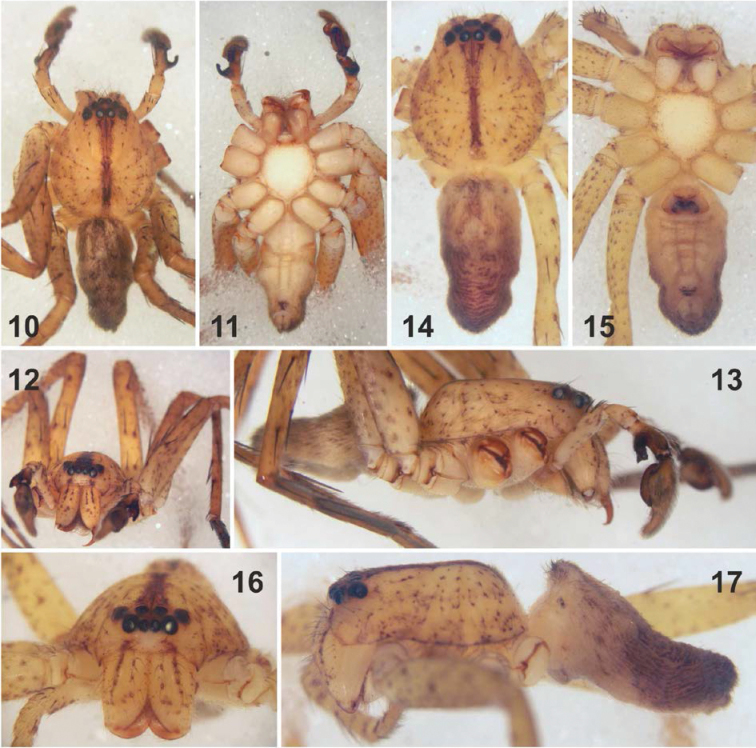
*Pseudopoda ashcharya* sp. n. from India, habitus of male (**10–13** holotype) and female (**14–17** paratype) (**10, 14** dorsal **11, 15** ventral **12, 16** frontal **13, 17** lateral).

Colouration (Figs 10–13): Light yellowish brown with brown markings. DS dotted, with narrow dark longitudinal band running from PME to posterior end of fovea and slightly darker lateral margins as well as indistinct submarginal band. Sternum, labium, gnathocoxae and coxae ventrally pale yellowish without pattern. Chelicerae yellowish brown with two distinct longitudinal bands frontally and one indistinct band laterally, in distal half with dots. Legs spotted, femora with additional spine patches. OS dorsally with alternating dark and light bands in anterior half and paired patches in posterior half; laterally spotted; ventrally with dark triangle in front of spinnerets.

Female. DS length 4.0, width 3.4, anterior width 1.9, OS length 4.3, width 2.0. Eyes: diameters AME 0.19, ALE 0.32, PME 0.26, PLE 0.30; interdistances AME–AME 0.13, AME–ALE 0.05, PME–PME 0.15, PME–PLE 0.35, AME–PME 0.33, ALE–PLE 0.32, clypeus height at AME 0.49, at ALE 0.39. Spination: palp: 131, 101, 2121, 1014; legs: femur I–III 323, IV 321; patella I 001, III–IV 101; tibia I–IV 2026; metatarsus I–II 1014, III 2025, IV 3036. Metatarsus IV ventrally with double row of bristles along entire length and with patch of bristles instead distal spine, I–III with scopula, without bristles. Leg formula: 2413. Measurements of palp and legs: palp 5.4 (1.6, 0.8, 1.2, -, 1.8), leg I 15.4 (4.3, 1.8, 4.2, 3.7, 1.4), leg II 16.5 (4.7, 1.9, 4.5, 4.0, 1.4), leg III 12.7 (3.8, 1.5, 3.2, 3.0, 1.2), leg IV 15.8 (4.6, 1.5, 4.0, 4.2, 1.5). Promargin of chelicerae with 3 teeth, retromargin with 4 teeth; cheliceral furrow with 20–21 denticles in slightly elongated patch close to anterior teeth; chelicerae with 1 bristle close to retromargin of fang base. Palpal claw with 6 teeth.

Copulatory organ as in diagnosis (Figs 5–9). Epigyne wider than long, epigynal field without distinct anterior bands. Lateral lobes rounded at their posterio-lateral margin, protruding distinctly over epigastric furrow at about half of their length, with pockets between lateral parts and epigastric furrow; fused along the median line with indistinct external ledges and internal “fusion bubbles” (Jäger and Krehenwinkel 2015). Internal duct system with first winding bulging laterally, spermathecae situated postero-laterally. Fertilisation duct arising posteriorly from spermathecae, apical end antero-mediad.

Colouration (Figs 14–17): As in male but inner frontal band on chelicerae developed as row of dots, lateral band lacking.

##### Distribution.

**Figure 18.**
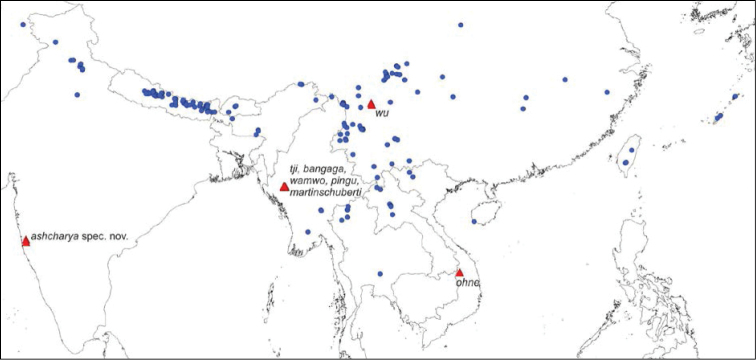
Distributional records of *Pseudopoda* species. Red triangles – species without conductor; blue circles – species with conductor.

Known from two localities in the Western Ghats in India (Fig. 18).

## Discussion

*Pseudopoda* species have been recorded from South, East and the north-western part of Southeast Asia (Fig. 18). Until now seven out of 120 species are known to have the conductor reduced (Jäger et al. 2015, Jäger 2015, Logunov and Jäger 2015). They are distributed in Yunnan, China and Chin State, Myanmar, as well as in Central Vietnam (Fig. 18). The present species is known from two localities in Maharashtra State, India. These are about 1,400 km away from the nearest occurrence of congeners in the Himalaya and more than 2,000 km from the closest locality with conductor-less congeners in Myanmar. The question is how this isolated occurence can be explained. The geological history of the Indian West coast indicates that the Western Ghats have been formed 150 mya during the break-up of Gondwana and came into being around 100 to 80 mya. This period was suggested as the time when the basal split within the Sparassidae occurred (Moradmand et al. 2014) rather than a time of diversifying within single genera like *Pseudopoda* (< 50 mya: Moradmand et al. 2014). During the same period (circa), the Indian raft was introduced to Asia allowing a passage for exchange of species (Conti et al. 2002). Most of India (except part of northwest) was covered by humid forest continuous with forests of South-east Asia, receiving high rainfall during 18–11 mya. The arrival of drier climate 5 mya onwards wiped out this wet zone isolating Western Ghats and parts of Eastern Ghats from the south and south-east Asian wet zones (Karanth 2003: fig. 3). When looking for other today’s special criteria of the Western Ghats the annual precipitation of over 1000 mm might be one factor that could explain the isolated occurrence of *Pseudopoda ashcharya* sp. n. Regions east of the Western Ghats have less than 200 mm annual rainfall which might represent a barrier for species adapted to moist and humid conditions. For this scenario it seems likely that the new species is a relict of a previously wider distribution range of the genus. However, the dis-junction in the distribution of *Pseudopoda ashcharya* sp. n. and its congeners from India and neighbouring countries might also be an artefact arising from lack of extensive survey in central and west Indian states.

From the morphology, males of the new species are apparently close to *Pseudopoda* species recently described from the southern Chin State in Myanmar. The conductor of species from both localities is reduced. Jäger (2015) considered a dense brush of setae as potential functional surrogate structure. This brush occurs in *Pseudopoda ashcharya* sp. n. as well (Figs 1–2: B). Females, however, show a unique character within the entire genus: the lateral lobes are medially fused with “fusion bubbles” as indication for such an evolutionary event (Figs 5, 8: FB). Similarly, fused lateral lobes are known only from *Sinopoda* Jäger 1999 and to a lesser extent from *Bhutaniella* Jäger 2000. But the lack of other structures diagnostic for these latter two genera, such as for instance the epigynal pockets as well as the typical bifid and complex embolus in *Sinopoda* and *Bhutaniella* respectively make clear that the present species does not belong to either of these genera. The similarity of the male copulatory organ with those of other *Pseudopoda* species and the congruence of diagnostic characters according to the most recent diagnosis of the genus (Jäger et al. 2015) as well as similarities of other characters in the female copulatory organs and their congruence with diagnostic characters (Jäger 2001; here especially the course of the internal duct system as shown in fig. 82) let suggest placing the species in *Pseudopoda*.

## Supplementary Material

XML Treatment for Pseudopoda ashcharya
